# Social Innovation Toward a Meaningful Everyday Life for Nursing Home Residents: An Ethnographic Study

**DOI:** 10.3389/fpsyg.2021.666079

**Published:** 2021-11-25

**Authors:** Åshild Slettebø, Ragnhild Skaar, Kari Brodtkorb

**Affiliations:** Department of Health and Nursing Science, Centre for Caring Research – Southern Norway, Faculty of Health and Sport Sciences, University of Agder, Grimstad, Norway

**Keywords:** nursing, community of practice, culture change, nursing homes, qualitative method

## Abstract

**Background:** The literature shows that innovation, which includes culture change, may be important to create a meaningful everyday life for nursing home residents. However, there is a gap in how social innovation practices may contribute to this. The theoretical discourse for the study is person-centered care.

**Aim:** The main aim was to explore phenomena within social innovation that can contribute to improving nursing home residents’ everyday lives.

**Design and Method:** This study uses an ethnographic design with observations and interviews in two nursing homes in Southern Norway.

**Findings:** The main theme was that social innovation within working practices in nursing homes includes phenomena that contribute to a meaningful everyday life for the residents. This main theme includes five subthemes: (1) opening the nursing home to the surroundings; (2) expanding and strengthening the community of practice; (3) facilitating customized activities; (4) ensuring sufficient nutrition and facilitating enjoyable mealtimes; and (5) preventing unrest and disturbing behavior.

**Conclusion:** The study reveals that innovation practices grounded in person-centered care in nursing homes may contribute to opening the nursing home to the community and establishing a common community practice for all members of the nursing home. This enables residents to experience meaningful everyday life through customized activities, sufficient nutrition, and a pleasant milieu during mealtimes. Disturbing behavior is also prevented, making it possible to promote meaningful lives in nursing homes.

## Introduction

Statistics Norway (2020) reports that there were 48,889 residents in nursing homes in Norway at the end of 2019. Of these, 32,105 were residents in long-term facilities and 9,784 were residents in short-term facilities (Statistics Norway, 2020). In 2018, there were approximately 70,000 people living in Norway who were diagnosed with dementia, and more than 80% of the residents in nursing homes had been diagnosed with dementia (Ministry of Health and Care Services, 2018). In Norway and in Western society more generally, there has been a shift in focus in nursing homes. Although physical care used to be the most prominent focus, recently, there has been a shift toward a holistic view of the person and person-centered care, seeing the whole person and their physical, psychological, spiritual, and social dimensions. There has been a change from more passive care to active care, with meaningful activities highlighted as important in long-term care (Ministry of Health and Care Services, 2018). In Norway, “teaching nursing homes” have been established with the intention of enhancing and developing the quality of care provided in nursing homes (Ministry of Health and Care Services, 2006). In the current study, two nursing homes were used as the sites for an observational study design looking at social innovative practices for making everyday life meaningful for the residents.

## Background

The importance of meaningful everyday life for nursing home residents has been emphasized (Ministry of Health and Care Services, 2009). To have a meaningful life is important for all people, regardless of their age or living situation. When an old person moves into an institution, they become dependent on healthcare personnel for possibilities to create meaning in their everyday life. This is what nursing is about (Travelbee, 1971). However, how to facilitate meaning in life for nursing home residents has not been fully explored, but it is important for residents to experience dignity in everyday life when living in an institution (Kinnear et al., 2015; Slettebø et al., 2017).

There is a need for a cultural change in nursing homes that includes the ethics of care. In the literature, some cultural changes in aged-care facilities have been found to be crucial. These include changes, such as more individualized care, the facilitation of more meaningful relationships, better opportunities for participation in life roles more generally, and the possibilities for the residents to experience a new sense of belonging (Andrew and Ritchie, 2017).

Organizational culture shapes nursing home care. Culture change in nursing homes is a broad-based effort to make nursing homes less institutional and more homelike, patient- and family-centered while creating workplace practices that empower staff (Lima et al., 2020). An integrative review (Duan et al., 2020) of the empirical evidence of the effects of such cultural changes in nursing homes shows a positive trend in terms of residents’ quality of life, satisfaction, and autonomy.

In the care for the elderly, there is often an imbalance in power among caregivers and care receivers. However, relational practices are considered a two-sided coin, here with a focus on solidarity and care (Jennings, 2018). To have a good quality of life and a meaningful life, relationships are important for residents in long-term wards in nursing homes. To focus on solidarity and care, caregivers should provide equal respect for the residents’ rights and dignity, as well as provide health services. This contributes to “the actualization of potential flourishing for each and all” (Jennings, 2018, p. 553). Sources of meaning in life for older psychologically frail people are financial security, meeting basic needs, and personal relations (Hoeyberghs et al., 2019). This is the first study of the association between meaning in life and psychologically frail older people, showing the sources of meaning in life that should be met by caregivers. Residents in nursing homes may be seen as psychologically frail because most suffer from dementia. Thus, meeting basic needs and establishing solid personal relationships with residents could improve meaning in life for residents.

Meaningfulness and meaning-making are addressed in both psychological (King et al., 2006, 2016; Park, 2010; George and Park, 2016; Wong, 2017; Martela et al., 2018; Crego et al., 2020) and nursing research (Glaw et al., 2017). Meaning-making is considered to have three different meanings: “(a) cognitive processes of attribution and appraisal, (b) creative work of employing one’s gifts to make a useful contribution, and (c) narrative process of constructing a personal story to make sense of an event or one’s life” (Wong, 2017, p. 86). However, an important question regarding meaning-making is what is really meaning-making for this particular person and under what circumstances. Then, one may discuss particular types of meaning-making and if different meanings of the concept are made helpful and why (Park, 2010). Also, meaningfulness has two different aspects, mainly the cognition which is making sense of life and motivation which is to have a sense of purpose in life (Wong, 2017).

The importance of addressing meaningfulness is emphasized by pointing at the psychopathologies that may arise of meaninglessness, such as depression, anxiety, addiction, aggression, hopelessness, apathy, lower levels of well-being, physical illness, and suicide (Glaw et al., 2017). By focusing on positive affect and relationships, especially with family, healthcare personnel can reduce these psychopathologies and enhance meaningfulness in life with positive well-being, happiness, and better coping with stressful events (King et al., 2006, 2016; Glaw et al., 2017; Crego et al., 2020). Another way to make meaning in life and experience meaningfulness is found to be experiencing autonomy and competence, as well as satisfaction of relatedness and beneficence (Martela et al., 2018).

This is interesting findings also regarding older people in nursing homes as meaningfulness is important for thriving. Phenomena that may contribute to residents’ experiences of meaningfulness is found to be personal relationships, especially with family, but also to create moments of personal growths may increase meaning in life for older people also lacking cognitive difficulties (Dewitte et al., 2019). In a recent article, the components of meaning: coherence, purpose, and significance, were studied in a sample of residents lacking Alzheimer’s disease (Dewitte et al., 2021). The participants had a felt coherence rather than a cognitive one, and the purpose was not future-oriented any longer. However, the most important phenomenon for them to experience meaning in life were relationships and family. This understanding of meaning in life is consistent with the three components comprehension, purpose, and mattering (George and Park, 2016).

Social innovation is important to facilitate a culture that underpins a meaningful everyday life for nursing home residents. Innovation in the public sector, of which nursing homes may be considered a part, is of interest when the intentions of nursing homes change, as they have lately. Public sector innovation is defined as follows:

*Public sector innovation is about new ideas that work at creating public value. The ideas have to be at least in part new (rather than improvements); they have to be taken up (rather than just being good ideas); and they have to be useful. By this definition, innovation overlaps with, but is different from, creativity and entrepreneurship* (Mulgan, 2007, p. 6).

This definition includes the notion that innovation implies something “new, useful, and utilized” (Wegener and Tanggaard, 2013). Pue et al. (2016, p. 10) define social innovation as follows:

*A new definition of social innovation, characterizing it as a process encompassing the emergence and adoption of socially creative strategies, which reconfigure social relations in order to actualize a given social goal*.

Innovation may be considered a way to explore and fulfill the mandate of nursing homes. This mandate implies that because residents spend the later part of their lives in nursing homes, it is important that these homes be experienced as affording a meaningful life for their long-term residents. Innovation in nursing homes can also impact staff perceptions; social support seems to be very important for the social climate among healthcare personnel in nursing homes and, thus, for job satisfaction and job motivation (Adams et al., 2017). This, in turn, impacts healthcare in nursing homes and the service given to residents.

Although perfection is impossible, improvement is not (Tronto, 2001), and care should take place in an environment where all parts engaged can contribute to the ongoing discussion on the best way to meet needs.

Theories of meaningfulness, as discussed by Narens (2002), concentrate on meaningfulness in behavioral sciences but take their starting point in mathematics as cornerstone for these theories. Research in behavioral sciences is discussed mainly with a focus on quantitative research methods, even though qualitative case studies are mentioned (Gravetter and Forzano, 2018). Thus, the main understanding of theories of meaningfulness in behavioral sciences is based on quantitative and experimental research.

The current study is qualitative and focuses on how social innovation through culture change can contribute to residents in nursing homes experiencing a sense of meaning in their daily life. However, meaning-making, as described in narrative therapy, may be relevant for residents in nursing homes, where meaning creation of events in life is seen as a way to cope (Kropf and Tandy, 1998; Fivush et al., 2017). This is relevant in person-centered care, so this framework is chosen as the theoretical discourse in the current study.

Thus, the present study’s theoretical discourse of person-centered care is a theory developed by the psychologist Tom Kitwood (Kitwood and Bredin, 1992; Kitwood, 1997). The aim of the theory is to prevent unhealthy sociopsychological patterns in institutions for older people. The theory has been further developed to be implemented in care institutions by McCormack (2004, 2010). It is often found as fundamental for cultural change toward accomplishing a meaningful life for residents in nursing homes (Brodtkorb et al., 2019). Person-centered care emphasizes the meaning of life and the resident’s life history as the basis for providing care for the resident both generally and especially for those residents suffering from a form of dementia. The unique persons living in the nursing homes and the knowledge and experiences of the staff are necessary for planning and implementing care and activities that create meaning-making for the residents. Kitwood (1997, p. 8) defines person-centeredness in the following way:

*…a standing or status that is bestowed upon one human being, by others, in the context of relationship and social being. It implies recognition, respect and trust*.

McCormack (2004, p. 33) argues that it is one’s moral personality that gives a person status as someone special. It is the moral attitude that implies humanity as personhood. Because of this moral personality, the “person has an intrinsic, inalienable, unconditional, and objective worth and dignity as person.” In a review article by McCormack (2004), person-centered nursing is shown to be based on four understandings of (a) being in relation, (b) being in a social world, (c) being in place, and (d) being with self. These understandings of person-centered nursing imply knowing the resident, and here, it is central to know the resident’s values, biography, and “negotiated relationship” with the resident.

The literature shows that innovation, which includes culture change, may be important to create improvements for nursing home residents. However, there is a gap in how social innovation practices may contribute to this improvement.

## Aim

The main aim was to explore phenomena within social innovation that can contribute to improving nursing home residents’ everyday lives.

## Design and Method

The current study has an ethnographic design, with observations and interviews conducted in two nursing homes in Southern Norway.

### Context

The nursing homes in the present study had resident departments consisting of single rooms spread over several units with a shared kitchen and living room. There was a separate resource department in one of the nursing homes for people with dementia and challenging behavior. There were day centers, a cafeteria, a hairdresser, and a podiatrist in the centers, as well as a memory room and large living room with stage facilities. On their webpages, the nursing homes expressed a wish to be hospitable and open centers where everyone respects each other and treats each other well.

In Norway, there is an established “Joy of Life Nursing Home” movement.^1^ This is a certificate arrangement for nursing homes that fulfill certain standards in terms of respecting residents’ existential, cultural, and psychosocial needs. The nursing homes included in the current study were certified as “Joy of Life Nursing Homes” and had been recertified several times, including during the period when the observations took place; this means that they were obliged to be innovative and develop qualitative good care to meet residents’ needs.

### Data Collection

Two researchers (RS and KB) were participating observers at the two nursing homes. Field observations were performed for a total of 320h spread over a period of 16months from fall 2016 to fall 2018. This resulted in 85 pages of single-spaced field notes in font size 12. The field notes were recorded and transcribed in full. The two researchers observed one nursing home each; they were observing in the common area at the nursing homes, but were not participating in care activities that took place in the private rooms of the residents. Sometimes, they were talking with the staff to get a deeper understanding of what was happening in some situations. Some days, they observed more generally what happened at the living rooms or day center, and some days, they followed particular nurses to observe how they performed daily activities in common areas. Two in-depth interviews were conducted with nursing home leaders at the start of the observation period. The interviews were recorded and transcribed verbatim. The interviews lasted between 68 and 70min. The role of the interview was to deepen the understanding of the philosophy of the nursing home leaders in front of the data collection through the field observations. Thus, the main data came from field notes from the observations. The transcriptions from the interviews were analyzed as text, together with the data from the field notes. In addition, two dialogue meetings were conducted with the staff of the two nursing homes at the beginning and after a period of 2years (a total of four meetings), and the minutes were added to field notes and analyzed accordingly.

### Analysis

A thematic qualitative analysis (Braun and Clarke, 2006) was performed by all three researchers, leading to a main theme and five subthemes. All data from the two interviews, the field notes, and the minutes from the dialogue meetings were merged together as one text and analyzed as a whole. The researchers followed six steps. First, we familiarized ourselves with the data by reading the entire text several times before we met and started the initial analysis. Then, we had an impression of what the themes in the text were about but not having named the themes yet. Second, we generated initial codes, such as “visit from kindergarten and residents were touched and happy,” “cleaners actively participating in the staffs’ effort of making meaningful days for the residents thus being part of the community of practice,” “there is a training trail with varied surface giving opportunity for residents to walk outside,” “there are different animals visiting the nursing home where the residents can enjoy meeting animals if they like,” “residents’ experience of joy when eating newly baked bread,” or “resident had challenging behavior and mapping helped finding information.” Third, we searched for more abstract themes that were then coded and collated. These codes were “social innovation,” “nutritional status,” “open nursing home,” “meaning in life,” and “meaningful activities.” The analysis process is described in a table (see Table 1). Next, we reviewed the themes before defining and naming five different subthemes: (1) opening the nursing home to the surroundings; (2) expanding and strengthening the community of practice; (3) facilitating customized activities; (4) ensuring sufficient nutrition and facilitating enjoyable mealtimes; and (5) preventing unrest and disturbing behavior; and one main theme “Social innovation within working practices in nursing homes includes phenomenon that contribute to a meaningful everyday life for the residents” that addressed the research question. The final and sixth step was reporting the findings.

**Table 1:** Examples of the analysis process.

Quotation with meaning unit	Category	Code	Subtheme	Main theme
A group of children from a kindergarten come singing through the corridors, dressed as Lucia, and holding candles. One of the residents is clearly touched and happy about the visit (Field notes).	Visit from kindergarten Residents touched and happy	Open nursing home Meaning in life	Opening the nursing home to the surroundings	Social innovation within working practices in nursing homes includes phenomenon which contribute to a meaningful everyday life for the residents
One of the cleaners had fresh shrimp as her husband fished early in the morning today. She has distributed them to all departments. The pupil and the apprentice clean them at the Table. A male resident looks on, and we get a little conversation about fresh shrimp and shrimp fishing (Field notes).	Cleaners actively participating in the staffs’ effort of making meaningful days for the residents thus being part of the community of practice	Social innovation in practice Meaning in life	Expanding and strengthening the community of practice	Social innovation within working practices in nursing homes includes phenomenon which contribute to a meaningful everyday life for the residents
We have made a training trail where one can go on a varied surface, where there are pebbles, where there is bark, where there is gravel, to get a bit of that balance training. (…) We have chickens, cats, dogs, visiting dogs, birds such as budgies. (…) We have what we call a “sound shower” where you can go in, sit down, and listen to birds chirping, or you can choose a boat engine if you like, where you can hear the waves (Manager interview, nursing home 1).	There is a training trail with varied surface giving opportunity for residents to walk outside There are different animals visiting the nursing home where the residents can enjoy meeting animals if they like	Meaningful activities	Facilitating custom activities	Social innovation within working practices in nursing homes includes phenomenon that contribute to a meaningful everyday life for the residents
A patient expressed joy in “hot bread.” There is baked bread every morning in the canteen, which is brought to the department (Field notes).	Residents’ experience of joy when eating newly baked bread	Nutritional status Meaning in life	Ensuring sufficient nutrition and facilitating enjoyable mealtimes	Social innovation within working practices in nursing homes includes phenomenon that contribute to a meaningful everyday life for the residents
There was a patient who was challenging, and they somehow could not quite figure out how to help him. Then they mapped [by Dementia Care Mapping] him for a day and an evening and found quite a lot of interesting information (Manager interview, nursing home 2).	Resident had challenging behavior and mapping helped finding information (on how to prevent unrest)	Meaningful activities	Preventing unrest and disturbing behavior	Social innovation within working practices in nursing homes includes phenomenon that can contribute to a meaningful everyday life for the residents

### Ethics

The nursing homes included in the current study participated in a larger research project named “Social Innovation in Nursing Homes,” which was funded by The Norwegian Research Council (no. 256647). Because of the study aims and Norwegian legislation, which requires ethical approval only when health information is collected as part of the research, ethical approval was not required. However, the principles of the Declaration of Helsinki (World Medical Association, 2013) were followed. Observation only took place in communal areas and not in private or intimate situations or in the residents’ rooms; thus, privacy was respected. Participation in observation and interviews was voluntary and based on informed consent. The participants received information in advance, were able to withdraw at any time, and could refuse to be observed. The project was approved by the Norwegian Centre for Research Data (no. 49229).

## Findings

The main theme was that social innovation within the working practices in nursing homes includes phenomena that contribute to a meaningful everyday life for the residents. This main theme includes five subthemes: (1) opening the nursing home to the surroundings; (2) expanding and strengthening the community of practice; (3) facilitating customized activities; (4) ensuring sufficient nutrition and facilitating enjoyable mealtimes; and (5) preventing unrest and disturbing behavior.

Social innovation means that everything revolves around human resources, where everyone contributes and is included. While conversing with two nurse aides who had extensive experience both at the current nursing home and at other nursing homes, the researcher noted the following:

*The nurse aides had both worked at another nursing home before. These places have been more marked by practical work and the assignment of tasks. They thrive better here, where they feel that the focus every day is to make the days as good and pleasant for everyone as possible* (Field notes, p. 53).

Here, the nurse aides experienced the care culture and working environment as positive, solution focused, and innovative. The employees had positive attitudes toward change, as observed during the fieldwork and after changes had been implemented. The staff showed a value base that was realized in daily work focusing on person-centered dementia care.

*She says that she has previously had practice at another institution, and that what stands out about this nursing home is the focus on joy of life. The fact that each patient’s life history is recorded and available to her has helped her become more and more familiar with each resident. She notes that the daily life pleasure activities for the residents she follows up on every day encourage her to be active* (Field notes, p. 55).

The value base appears to have been founded on every employee knowing the residents, caring about them, and finding value in them.

### Opening the Nursing Home to the Surroundings

The first subtheme was “opening the nursing home to the surroundings.” The nursing homes were open institutions, meaning that they extended into the community and invited the community into the nursing homes to participate in activities. An open nursing home contributed to meaningful everyday life for the residents, employees, students, relatives, volunteers, and other visitors.

*A group of children from a kindergarten come singing through the corridors, dressed as Lucia, and holding candles. One of the residents is clearly touched and happy about the visit* (Field notes, p. 55).

The kindergarten near the nursing home came to visit the residents and included them in their activities. The residents also visited kindergartens and organizations; for example, bands had their meetings and rehearsals in the dining room of one of the nursing homes. On Constitution Day, the children marched through the garden of one of the nursing homes and visited the residents. Hence, there were fluid transitions between the nursing homes and the community at large.

There were clearly good intentions regarding taking care of the residents’ needs, but some conflicting considerations for the nurses and other healthcare personnel were observed. For example, sometimes, there were too few personnel at the unit to take care of all the residents’ needs. In addition, a lack of resources meant that it was not possible to attend to needs that should be met individually. One nursing home had its own resource ward with a special competence in dementia care. The employees from this ward were experts who conveyed their expertise broadly to other departments and employees, as well as to the other nursing home, the municipality, and relatives.

Each nursing home had a cafeteria open to all who wanted to visit for a cup of coffee or some food. It was used by the residents, relatives, visitors, and others from the community. Here, the residents could chat with new people or meet relatives or visitors. There were also meetings between different generations; kindergarten children and middle school children visited the nursing homes. Groups with accordion players came and had singing sessions or other types of concerts with the residents and others who wanted to participate. However, the children performed for the residents (entertainment), but it amounted to minimal interactions between generations rather than mutual activities:

*The student and I (the observer) are attending singing time with kindergarten kids at the day center. It is organized as a small concert where the children sing for the old and get applause and waffles. There is no further interaction between the kids and the residents* (Field notes, p. 27).

It was also noticed that there could be enhanced interaction between visitors and residents to further assist in meaning-making for the residents when visitors came to the nursing home. This led to the second subtheme about creating a community of practice at the nursing home.

### Expanding and Strengthening the Community of Practice

The second subtheme was “expanding and strengthening the community of practice” in the nursing home. One finding was that all the employees were important participants in making the day meaningful for the residents. Here, the manager included all the employees of the nursing home, from the chef, custodian, and cleaning personnel to the physician, physiotherapist, and nursing personnel. Everyone cooperated, all were needed, and a common culture was central to innovation. The manager emphasized that everyone had equal importance, and the staff were included in activities with the residents. This was important because the staff did activities together with the residents rather than for the residents. Absenteeism among the staff was low because everybody felt included and important for the well-being of both the residents and other employees. However, there were challenges in terms of the different workloads on the different wards, as one ward leader explained:

*She also says that over the years, this ward has received residents with more challenging behavior than the other ward at the nursing home, which has the same number of residents and equal staffing. (…) If individuals continue to criticize things that have already been decided, she sees it as the leader’s responsibility to point out to them how it impacts the community and the motivation of others to maintain a positive attitude toward change* (Field notes, p. 34).

This note shows the importance of the leader at each ward and their role in maintaining a positive attitude toward change and the work at the ward. The nursing homes cooperated with labor outreach and had persons with mild intellectual disabilities in service. These people did their jobs well and positively supplemented the staff. This was an element of the open and community-oriented nature of the nursing homes that served as a mutual advantage for both the employees and the rest of the staff. For example, a member of the cleaning staff brought newly fished shrimp to one meal for all residents and staff, including all the members of the staff in meals and nutrition:

*One of the cleaners had fresh shrimp as her husband fished early in the morning today. She has distributed them to all departments. The pupil and the apprentice clean them at the table. A male resident looks on, and we get a little conversation about fresh shrimp and shrimp fishing* (Field notes, p. 55).

As the note describes, each staff member, regardless of profession, experienced being included in meaning-making for the residents. Each member’s contribution was seen as important. Reflection meetings were organized on each ward, and competence development was systematically given attention, both in work schedules and through participation in external courses. Cooperation between all levels in the healthcare sector and transdisciplinary collaboration was important parts of seeing and using all resources to the best effect for the residents. The staff had regular meetings with the hospital staff, as well as with staff from home healthcare in the community. The physiotherapist and occupational therapist had their offices outside the nursing home and visited special residents when needed. This was experienced as challenging by several of the care personnel. Each nursing home had its own full-time physician who attended meetings with both the healthcare personnel at the nursing home and collaborating partners.

### Facilitating Custom Activities

The third subtheme was “facilitating custom activities.” The nursing homes in the current study were certified as “Joy of Life Nursing Homes.” Because of this, several activities were offered that were more structured and systematized. The staff at the nursing homes arranged parties for residents, relatives, and others from the community based on the seasons. For example, they had a summer party at the beginning of the summer to mark the new season. This was outside in the garden, with a barbecue in the sun. In addition, they arranged a new party when autumn came to mark the transition to the new season.

The nursing homes had also bought rickshaw bicycles and had hired young adults to bicycle the residents on tours around the nursing home area. They went to the city nearby and to the woods or the seaside. This was seen as place bonding and part of place attachment for the residents enhancing their well-being and quality of life. These bicycles were also made available to relatives to take residents for a ride if they wanted.

In our observations, we noted that the most well-functioning nursing home residents received activity tasks at the day center, while the rest received care in the wards, where quiet activities were the focus. As one leader described in an interview, there were many possibilities for activities:

*We have made a training trail where one can go on a varied surface, where there are pebbles, where there is bark, where there is gravel, to get a bit of that balance training. (…) We have chickens, cats, dogs, visiting dogs, birds such as budgies. (…) We have what we call a “sound shower” where you can go in, sit down, and listen to birds chirping, or you can choose a boat engine if you like, where you can hear the waves* (Manager interview, nursing home 1, p. 12–13).

Even though there were several possibilities for activities, there were also limitations on physical frameworks, but the staff found it important to stay focused on what they could achieve, not on the limitations. They focused on solutions. When talking to one of the experienced nurse aides, the researcher noted the following:

*She thinks that it is busier than before and that there is too little time to concentrate on meeting with the residents at their pace and on their premises. We are talking about the new garden which will officially open in two days. She thinks that it is fine, but at the same time not completely safe for the residents of the ward. The garden is on a steep slope, and there is a hill that is too steep for many residents* (Field notes, p. 33).

As the quotation shows, examples of limitations were that staffing was not ideal, the sensory garden was very steep and difficult to use for some of the residents, and there were resident rooms on the outskirts of the department. However, the staff did the best they could for the residents given the circumstances.

### Ensuring Sufficient Nutrition and Facilitating Enjoyable Mealtimes

The fourth subtheme was “ensuring sufficient nutrition and facilitating enjoyable mealtimes.” Systematic monitoring of nutritional status is important for the residents’ quality of care. On both the individual and system levels, the aim was to prevent and treat malnutrition, thereby ensuring a surplus of energy for the activities of daily living. As part of the focus on nutrition, the National Patient Safety Program was used (one of the themes is nutrition).

In the residents’ electronic health records, there used to be several points to mark regarding nutritional status. Through an innovation process, this was simplified to two categories: nutrition mapping and nutrition plan. This provided the staff with an overview of each resident’s weight and body mass index (BMI) on a certain date so that they could assess which residents were at risk because they had lost weight. The staff paid more attention to weight and BMI than they had before and made sure that the nutrition mapping was systematically done and that it resulted in a nutrition plan. They focused on meals, and both the nursing homes had changed the mealtime for dinner from about 1p.m. to 4p.m. and added a lunch meal between breakfast and dinner. The lunch was prepared on the wards by the healthcare personnel and nursing staff. It was discussed at meetings that this took some time to prepare, which some of the nursing staff would have preferred to spend with the residents.

However, the changes to the mealtimes largely functioned well. The staff noticed that the residents ate more and better and slept better after dinner time was moved. This change in mealtime improved the nutritional status for the residents in general, thus improving the resident’s energy to participate in meaningful and enjoyable activities. The nutrition plan was adjusted to prevent weight change – especially weight loss – in the residents:

*The electronic health record was simplified, and there are now only two categories regarding nutrition for each patient: (1) Nutrition Survey and (2) Nutrition Plan. Previously, patients were also weighed (mapped) and weight was registered, but measures were lacking when patients lost weight* (Field notes, p. 10).

After this change, the staff monitored weight loss specifically and adjusted nutritional status accordingly. This was important for the residents, so that they had the energy to participate in activities. Another important aspect of mealtimes was to make this time social and enjoyable. The staff participated and helped during mealtimes to create social encounters between the residents and staff.

*A patient expressed joy in "hot bread." There is baked bread every morning in the canteen, which is brought to the department* (Field notes, p. 4).

By offering newly baked bread, the nursing home staff helped residents enjoy eating breakfast more. However, it was observed that the staff prepared breakfast and did not let the residents prepare their own bread. This was done because it was easier for the staff and it did not take as long to serve the meal; however, this could be seen as a drawback in terms of the experience of a social meal for the residents. The dementia coordinator held regular meetings with the staff focusing on nutrition and other relevant topics:

*The dementia coordinator shows interest in nutrition. She asks how they plan and organize the meals, based on breakfast. The residents get ready-made food. She asks why. It turns out that the main reason for the ready-made food is the experience that if everyone sits together and helps themselves, there can easily be a lot of mess and chaos. Not everyone can do it properly and other residents can get annoyed. The dementia coordinator emphasizes that the staff play an important role in sitting at the table with the residents and coordinating the activities in the calmest way possible* (Field notes, p. 57).

Quality nutrition meant not only that meals consisted of food with the correct composition but also that meals should be meaningful and pleasant. Sufficient nutrition was about giving the residents surplus energy for other activities, both mentally and physically.

### Preventing Unrest and Disturbing Behavior

The fifth and last subtheme was “preventing unrest and disturbing behavior.” Restlessness and disturbing behavior can create challenges on the ward for employees and other residents. This was a special challenge in the wards where most of the residents suffered from dementia. However, the employees controlled violence and threat challenges with risk assessments and training in handling such behavior. The field notes reveal that the staff had separate meetings on how to handle violence and threat challenges. As part of handling violence and threats, the National Patient Safety Program was used. The staff discussed how to map threat challenges, who posed these threats, and what sort of threats they were. They experienced direct violence, both verbally and physically. They then discussed how to meet such threats in different ways and what measures to apply in different situations. A nurse manager explained the following in an interview:

*There was a patient who was challenging, and they somehow could not quite figure out how to help him. Then, they mapped [by Dementia Care Mapping] him for a day and an evening and found quite a lot of interesting information* (Manager interview, nursing home 2, p. 15).

Some of the methods consisted of training in theory and legislation, as well as how to relate in practically challenging situations and prevent such situations from occurring. The focus was on establishing trust between the residents and staff; they tried to provide for restless residents by allowing them to go outside and be followed only *via* GPS. They were not locked in the ward but could walk freely when they wanted. This was done to reduce feelings of being place-confined and thus reduce stress and anxiety and enhance place attachment to safeguard well-being and quality of life for the residents. These GPS arrangements were cleared with the residents, relatives, and physicians. Using GPS allowed the residents who was eager to walk and were suffering from restlessness to walk freely, thus enjoying activities that were meaningful to them. This led to a sense of freedom where they could walk alone, independent of staff, and to a feeling of freedom from visible surveillance, even though they knew they were tracked by the GPS.

Dealing with challenging behavior or restlessness was sometimes difficult when communication was impeded:

*The nurse leader’s experience was that residents accept employees with different looks or skin color. But it is a challenge that some employees do not master the Norwegian language well enough to communicate adequately with the residents. In a sheltered department with a lot of behavioral challenges, it is of great importance to be able to communicate clearly* (Field notes, p. 20).

This was considered when hiring new staff members, here with competence in Norwegian language being emphasized. There had been some staff absenteeism because of violence but not a lot. However, the employees reported that it was exhausting to work in an environment where threats or abuse were possible. This was also a problem for the other residents. Thus, it was important to prevent such behavior to promote a meaningful everyday life for all residents. The nurses met with the dementia coordinator and discussed how to handle situations of violence:

*There are many conditions that carers could not change (for example, diagnosis, older people), and other conditions that carers could change (provide good environmental measures, do something about the mental healthcare environment, document patient history, try to see the situation from the resident's point of view). Furthermore, one can usually try to assess what triggers a person. Nurses can also allow for extra time in the situation and be aware of the way in which they communicate in order to calm the situation* (Field notes, p. 17).

The dementia coordinator in the municipality met and talked with the nursing staff regularly to give advice on how to manage disturbing behavior from the residents suffering from dementia. Through different means, they managed to reduce and prevent this, thus promoting a meaningful everyday life for both the restless residents and their coresidents.

## Discussion

Our main aim was to explore phenomena within social innovation that can contribute to improving nursing home residents’ everyday lives. As a subtheme, we found that an open nursing home was an institution that made it possible for residents to experience meaningful everyday lives. This contrasts the “total institution” described by Erving Goffman in the 1960s (Goffman, 1961). Goffman (1961) describes the “total institution” as an institution that regulates and maintains the behavior of the patients and healthcare workers in a predictable and controlled way, such that they are “institutionalized” by knowing their social role within the institutional frames. In addition, a “total institution” is characterized by being separated from the surrounding environment; it is self-contained, with its own routines and frames. The risk of institutionalization and confinement for elderly is discussed by Ramkissoon (2020a). Ramkissoon (2020a) defines place confinement as perceived threat to mobility and affecting physical immobility where residents are restricted to their place of residence. Where residents’ physical activity is limited or restricted, it may result in feelings of dependency and impact feelings of well-being and quality of life (Ramkissoon, 2020a,b, 2021). However, place attachment may be fostered as a positive relationship to the environment. Ramkissoon et al. (2013, p. 554) defines place attachment as the bonding people share with places and consists of place dependence, place identity, place affect, and place social bonding. There has been a change in the ideologies of nursing homes from being closed and self-sufficient to being open and inviting the community into the institution. Thus, if healthcare personnel could help residents with enhancing feelings of place attachments to the environment surrounding the nursing home, it may impact their health, well-being and quality of life. Ramkissoon (2021) recommends promoting place affect interventions. Collaboration between residents, relatives, neighbors, and healthcare personnel opened up the institutions and the possibilities for residents to participate in the community as they had before they moved into nursing homes. This collaboration between residents and the community was also shown to be positive in Andrew and Ritchie’s research (Andrew and Ritchie, 2017).

We found that the residents needed different activities, and the staff at the participating nursing homes respected this. The healthcare personnel customized activities for each resident. Some of the residents were taken on tours with a rickshaw bike, while others were taken for small walks in the sensory garden; this depended on the resident’s capacity and motivation for activities. Some residents spent their time at the day center, where there were more activities, while others sat in the daily room or balcony on the ward, where it was quieter and there were fewer activities. This could be experienced as “slow nursing” (Lillekroken, 2020), where there is time to rest and have quiet time. This also gives healthcare personnel time for the residents to do the activities at a slower pace. This customization helped provide activities that could give the residents meaningful lives. The nursing homes were also customized for the residents to participate in different activities. However, there were some hindrances, as mentioned in the findings regarding the steep sensory garden. Even though the staff commented that this was not ideal, they did their best with the situation. The necessity of adjusting the surroundings of nursing homes has been pointed out in other research. Nordin et al. (2017) found that adjusting the environment at the nursing home was important so that the residents could do activities on their own. However, we found that the activities were not something that the residents were forced to participate in but instead something that they enjoyed participating in and that gave their lives meaning. This was in line with the “Joy of Life Nursing Home” movement, as well as with the research on dignity for residents in nursing homes (Shotton and Seedhouse, 1998; Brodtkorb et al., 2017; Slettebø et al., 2017; De Vriendt et al., 2019).

Introducing meaningful activities may contribute to establishing relationships and enhancing residents’ social lives (Cialdini and Goldstein, 2004; Cohen and Janicki-Deverts, 2009; De Vriendt et al., 2019). De Vriendt et al. (2019) provide a “because activities should be meaningful” approach with four dimensions: getting to know each other; self-prioritized goals; plan and actions; and evaluation and outcome. In a pilot study, the authors find that meaningful activities of daily living were enabled by a participatory client-centered approach, improving satisfaction and social life, and decreasing medication use in long-term care facilities (De Vriendt et al., 2019). Meaningful lives in nursing home residents may be fostered by focusing on positive affect and relationships, especially with family and to do activities together. By fostering resident’s positive affect the healthcare personnel can promote affective ties to a place for the resident such as the environment surrounding the nursing home, and thus support health, well-being and enhance quality of life for the residents (Ramkissoon, 2020a,b, 2021). Healthcare personnel can enhance meaningfulness in life with helping to create positive well-being, happiness, and better coping in residents (King et al., 2006, 2016; Glaw et al., 2017; Crego et al., 2020). This may be important for actually offer person-centered care to nursing home residents which is emphasized in the current study. Hauge and Heggen (2008) describe nursing homes as homes, explaining how common rooms, such as the dining room, can be used to enhance socialization and, thus, meaningfulness in everyday life.

This is in line with the theoretical concept of person-centered care in nursing homes. The focus on the resident’s life history is fundamental both in the person-centered care theory and in the “Joy of Life Nursing Home” movement, in which both the represented nursing homes participated in. The findings in the current study expand the understanding of the theory of person-centered care that a nursing home that is open toward society and expanding the community of practice where customized activities are facilitated were an important part of improving culture change and implementing person-centered care. In addition, it aligns with the understanding of person, which is outlined by McCormack (2004) and the four understandings of person-centeredness of Kitwood (1997) and McCormack (2004) of being in relation, being in a social world, being in place and being with self. Another aspect of person-centered care that is new knowledge from the current study expanding the understanding of person-centered care, is mealtime and meals that were enjoyable and customized for each individual resident, which also helped prevent unrest and disturbing behavior together with special care for each resident and their behavioral challenges.

During our observations, it was promising to see some interactions across generations, such as when kindergarten children or school students visited the nursing homes. This was the focus of the study of Gundersen and Slettebø (2016). However, we observed some activities in which the residents did not always participate fully; the residents watched the children and did not relate to them. There were other interactions that the residents enjoyed, such as when choirs or musicians performed concerts and the residents sang along. However, it may be a challenge to conduct these types of interactions in a way that engages all participants equally (Jennings, 2018). A secondary analysis of nursing home data showed that introducing a café in aged-care facilities contributed to transforming both the physical and social environment (Andrew and Ritchie, 2017). Social support seems to be very important for the social climate among healthcare personnel in nursing homes and, thus, for job satisfaction and job motivation. This, in turn, has an impact on healthcare services in nursing homes and the services given to residents (Adams et al., 2017). The need for meet basic needs and personal relations (Hoeyberghs et al., 2019) is shown for psychologically frail older people and should be considered when giving person-centered care to residents in nursing homes.

In the current study, we found that mealtime at the nursing homes had been changed so that dinner would be served later in the afternoon rather than at noon. This may be described as social innovation, as defined by Pue et al. (2016), where change is a process of socially creative strategies that reconfigure social relations. The innovation improved the dietary status of the residents because they ate better and more. It also afforded time for meaningful activities in the morning before lunch and dinner. There is also evidence in the literature of the positive effects of changing dining and mealtimes (Robinson and Gallagher, 2008). Changing the mealtimes allowed for more time in the morning to participate in meaningful activities. It could be relevant for the residents to experience meaning in life understood as comprehension, purpose, and mattering as discussed by George and Park (2016). In addition, it offered more time between breakfast and dinner, so the nutritional status of the residents improved, allowing them more energy to participate in activities that made the days more meaningful. This could be seen in the participation that occurred when children from kindergarten came to visit or allowing the residents themselves to go out on tours with bicycles. Changing dining services may be important in cultural changes in nursing homes (Robinson and Gallagher, 2008). Mealtime is considered important for nutritional status and socialization because residents should be allowed to make some choices regarding when, what, and where to eat and with whom. This is in line with the findings of Chwang (2012), who discusses issues of malnutrition in older people. One suggestion is to improve the quality of food services in healthcare facilities to improve mealtime experiences. In addition, it is important to understand the physiological changes in older persons and the need for nutritional knowledge to provide adequate nutrition care for the aged. By establishing a café in the nursing home, it is possible to enhance mealtimes in terms of individualizing care and socializing, providing the possibilities for new relationships and “to meet with staff, old friends, families, and members of the community” (Andrew and Ritchie, 2017, p. 42). In Norway, the government has invited nursing homes to establish kitchens in nursing homes instead of larger units outside the nursing homes (Ministry of Health and Care Services, 2018). This is a new policy in which a homelike atmosphere should be prioritized by building a kitchen in each nursing home. This is also in line with the experiences of other countries (Andrew and Ritchie, 2017).

The included nursing homes used GPS to prevent restlessness in residents who were uneasy and were calmed by walking. They did not have many fences and restrictions but let the persons wander as they wanted with GPS tracking. As our findings show, the use of GPS tracking allowed the residents to walk more freely without needed the staff to follow. For many of the residents, the walk outside gave meaning to their life and were enjoyable activities that made it easier to manage restlessness. To increase meaning in life for persons with cognitive difficulties family relationships and moments of personal growth is found to be important (Dewitte et al., 2019). To have the opportunity to walk freely may be such moments enhancing experiences of meaningfulness. This again may be seen as a new dimension of person-centered care. This is supported by the research of Ramkissoon (2020a) who finds that residents with pro-social behavior have reduced mental distress. Instead, such behavior fosters attachment, belongingness, trust, and life satisfaction and that activities in the nature help reduce anxiety and distress. However, there are ethical issues involved in tracking residents, and these were discussed with the residents, their relatives, and physicians before implementation. It was found to be a less-intrusive intervention than physically restricting wandering persons who wanted to go for long walks. This is in line with ethical recommendations in the literature (Landau and Werner, 2012; Hofmann, 2013). Because of the increasing number of older people in society, a welfare technology solution has been suggested. The ethical aspects of welfare technology are discussed in the literature (Landau and Werner, 2012; Hofmann, 2013). There are some challenges, such as alienation from human-to-human relationships. There could be different goals among different stakeholders. Residents might want to walk freely without being tracked, and if there are not enough staff to follow them, GPS would be the preferred intervention. This challenges confidentiality and privacy for the residents and poses problems when it comes to respecting dignity and vulnerability. Therefore, the literature recommends open discussions between residents and relatives, together with healthcare personnel, before implementing GPS and other welfare technologies in the care of residents diagnosed with dementia (Landau and Werner, 2012; Hofmann, 2013).

### Methodological Considerations

We believe that the current study meets the criteria for trustworthiness, confirmability, credibility, dependability, and transferability (Lincoln and Guba, 1985; Graneheim and Lundman, 2004). The study took place at two typical medium-size Norwegian nursing homes, which were considered innovative and accepted the researchers’ request to study their practices. This is in line with the criteria of confirmability and credibility. The researchers were familiar with nursing home practices and were open to what had happened in the setting. They knew the context because they were nurses and nurse lecturers who had been at nursing homes before but were not well known at the nursing homes in the study. However, there is a limitation: Observation took place only in communal areas, not in the residents’ rooms. The analysis was performed by the two observers and a researcher who did not participate in the observations, so we had both an inner and outer view of the data during the analysis. The research team discussed the analysis until agreement on the themes was reached. We have described the context and research process to the best of our ability so that transferability is possible; thus, the demand for trustworthiness is met. However, it must be considered that transferability to other contexts and countries may vary because of the different healthcare systems.

## Conclusion and Implications for Practice and Further Research

The current study has revealed that innovation practices toward holistic and person-centered care in nursing homes may promote quality improvement in nursing homes. Such practices contribute to opening the nursing home to the community, involving the whole facility and the surroundings, thereby establishing a community of practice for all members of the nursing home. This enables residents to experience a meaningful everyday life through customized activities, sufficient nutrition, and a pleasant milieu during mealtimes. By preventing behavior disturbances, it is also possible to promote meaningful lives in nursing homes. The current study adds to the theory of person-centered care that family relationships and moments of personal growth by fostering place attachment with the opportunity to walk freely, mealtime and meals that were enjoyable and customized for each individual resident, and helping to create positive well-being, happiness, and better coping in residents enhance experiences of well-being and quality of life by place bonding and meaningfulness. This again may be seen as new dimensions of person-centered care. In line with Ratwani (2017) suggesting that psychologists may support healthcare personnel with implementing Electronic Health Records, we suggest that the current study may imply that psychologist may support healthcare personnel with culture change in nursing homes when implementing these findings for enhancing meaningfulness and meaning-making for nursing home residents. Future research should look at how innovative practices and a more systematic opening of the nursing home to the community would foster homelike and meaningful stays in nursing homes.

## Data Availability Statement

The raw data supporting the conclusions of this article will be made available by the authors, without undue reservation.

## Ethics Statement

Ethical review and approval was not required for the study on human participants in accordance with the local legislation and institutional requirements. The patients/participants provided their written informed consent to participate in this study.

## Author Contributions

ÅS contributed to the study design and drafted the manuscript. RS and KB performed the data collection, and contributed to critical revisions and intellectual content during the review process and manuscript development. ÅS, RS, and KB performed the analysis. All authors contributed to the article and approved the submitted version.

## Funding

This research was funded by the Norwegian Research Council (no. 256647).

## Conflict of Interest

The authors declare that the research was conducted in the absence of any commercial or financial relationships that could be construed as potential conflicts of interest.

## Publisher’s Note

All claims expressed in this article are solely those of the authors and do not necessarily represent those of their affiliated organizations, or those of the publisher, the editors and the reviewers. Any product that may be evaluated in this article, or claim that may be made by its manufacturer, is not guaranteed or endorsed by the publisher.

## Footnotes

^1^ https://livsgledeforeldre.no/livsglede-for-eldre-engelsk/

## References

[ref1] Adams J. Verbeek H. Zwakhalen S. M. G. (2017). New models of care in residential long-term care: the impact of organizational innovations in nursing homes on staff perceptions: a secondary data analysis. J. Nurs. Scholarsh. 49, 54–62. doi: 10.1111/jnu.12271, PMID: 28094906

[ref2] Andrew A. Ritchie L. (2017). Culture change in aged-care facilities: a Café’s contribution to transforming the physical and social environment. J. Hous. Elder. 31, 34–46. doi: 10.1080/02763893.2016.1268557

[ref3] Braun V. Clarke V. (2006). Using thematic analysis in psychology. Qual. Res. Psychol. 3, 77–101. doi: 10.1191/1478088706qp063oa

[ref4] Brodtkorb K. Skaar R. Slettebø Å. (2019). The importance of leadership in innovation processes in nursing homes: an integrative review. Nord. J. Nurs. Res. 39, 127–136. doi: 10.1177/2057158519828140

[ref5] Brodtkorb K. Skisland A. V. S. Slettebø Å. Skaar R. (2017). Preserving dignity in end-of-life nursing home care: some ethical challenges. Nord. J. Nurs. Res. 37, 78–84. doi: 10.1177/2057158516674836

[ref6] Chwang L.-C. (2012). Nutrition and dietetics in aged care. Nutr. Diet. 69, 203–207. doi: 10.1111/j.1747-0080.2012.01617.x

[ref7] Cialdini R. B. Goldstein N. J. (2004). Social influence: compliance and conformity. Annu. Rev. Psychol. 55, 591–621. doi: 10.1146/annurev.psych.55.090902.142015, PMID: 14744228

[ref8] Cohen S. Janicki-Deverts D. (2009). Can we improve our physical health by altering our social networks? Perspect. Psychol. Sci. 4, 375–378. doi: 10.1111/j.1745-6924.2009.01141.x, PMID: 20161087 PMC2744289

[ref9] Crego A. Yela J. R. Gomez-Martinez M. A. Karim A. A. (2020). The contribution of meaningfulness and mindfulness to psychological well-being and mental health: a structural equation model. J. Happiness Stud. 21, 2827–2850. doi: 10.1007/s10902-019-00201-y

[ref10] De Vriendt P. Cornelis E. Vanbosseghem R. Desmet V. Van de Velde D. (2019). Enabling meaningful activities and quality of life in long-term care facilities: the stepwise development of a participatory client-centred approach in Flanders. Br. J. Occup. Ther. 82, 15–26. doi: 10.1177/0308022618775880

[ref11] Dewitte L. Schellekens T. Steger M. F. Martela F. Vanhooren S. Vandenbulcke M.. (2021). What can we learn about the concept of meaning in life from older adults with Alzheimer’s disease? A directed content analysis study. J. Happiness Stud. 22, 2845–2871. doi: 10.1007/s10902-020-00351-4

[ref12] Dewitte L. Vandenbulcke M. Schellekens T. Dezutter J. (2019). Sources of well-being for older adults with and without dementia in residential care: relations to presence of meaning and life satisfaction. Aging Ment. Health 25, 170–178. doi: 10.1080/13607863.2019.1691144, PMID: 31729244

[ref13] Duan Y. Mueller C. A. Fang Y. Tally K. M. (2020). The effects of nursing home culture change on resident quality of life in U.S. nursing homes: an integrative review. Res. Gerontol. Nurs. 13, 210–224. doi: 10.3928/19404921-20200115-02, PMID: 31968121

[ref14] Fivush R. Booker J. A. Graci M. E. (2017). Ongoing narrative meaning-making within events and across the life span. Imagin. Cogn. Pers. 37, 127–152. doi: 10.1177/0276236617733824

[ref15] George L. S. Park C. L. (2016). Meaning in life as comprehension, purpose, and mattering: toward integration and new research questions. Rev. Gen. Psychol. 20, 205–220. doi: 10.1037/gpr0000077

[ref16] Glaw X. Kable A. Hazelton M. Inder K. (2017). Meaning in life and meaning of life in mental health care: an integrative literature review. Issues Ment. Health Nurs. 38, 243–252. doi: 10.1080/01612840.2016.1253804, PMID: 27929687

[ref17] Goffman E. (1961). Asylums: Essays on the Condition of the Social Situation of Mental Patients and Other Inmates. USA: Vintage Books.

[ref18] Graneheim U. H. Lundman B. (2004). Qualitative content analysis in nursing research: concepts, procedures and measures to achieve trustworthiness. Nurse Educ. Today 24, 105–112. doi: 10.1016/j.nedt.2003.10.001, PMID: 14769454

[ref19] Gravetter F. J. Forzano L.-A. B. (2018). Research Methods for the Behavioral Sciences. 6th Edn. Boston: Cengage Learning, Inc.

[ref20] Gundersen E. D. Slettebø Å. (2016). Evaluering av et generasjonsoverskridende program med helsefremmende forankring: hvilke faktorer har bidratt til programmets vedvarende eksistens? [Evaluation of a cross-generational program based on promotion of health: which factors have contributed to the continued existence of the program?]. Nordisk Sygeplejeforskning 6, 6–19. doi: 10.18261/issn.1892-2686-2016-01-02

[ref21] Hauge S. Heggen K. (2008). The nursing home as a home: a field study of residents’ daily life in the common living rooms. J. Clin. Nurs. 17, 460–467. doi: 10.1111/j.1365-2702.2007.02031.x, PMID: 18205679

[ref22] Hoeyberghs L. J. Verté E. Verté D. Schols J. M. G. A. De Witte N. (2019). The importance of sources of meaning in life of community dwelling psychologically frail older people. Work. Older People 23, 65–76. doi: 10.1108/WWOP-01-2019-0001

[ref23] Hofmann B. (2013). Ethical challenges with welfare technology: a review of the literature. Sci. Eng. Ethics 19, 389–406. doi: 10.1007/s11948-011-9348-1, PMID: 22218998

[ref24] Jennings B. (2018). Solidarity and care as relational practices. Bioethics 32, 553–561. doi: 10.1111/bioe.12510, PMID: 30264873

[ref25] King L. A. Heintzelman S. J. Ward S. J. (2016). Beyond the search for meaning: a contemporary science of the experience of meaning in life. Curr. Dir. Psychol. Sci. 25, 211–216. doi: 10.1177/0963721416656354

[ref26] King L. A. Hicks J. A. Krull J. L. Del Gaiso A. K. (2006). Positive affect and the experience of meaning in life. J. Pers. Soc. Psychol. 90, 179–196. doi: 10.1037/0022-3514.90.1.179, PMID: 16448317

[ref27] Kinnear C. Victor C. Williams V. (2015). What facilitates the delivery of dignified care to older people? A survey of health care professionals. BMC Res. Notes 8:826. doi: 10.1186/s13104-015-1801-9, PMID: 26710882 PMC4693419

[ref28] Kitwood T. (ed.) (1997). “On being a person,” in Dementia Reconsidered: The Person Comes First (Milton Keynes: Open University Press), 7–19.

[ref29] Kitwood T. Bredin K. (1992). Towards a theory of dementia care: personhood and well-being. Ageing Soc. 12, 269–287. doi: 10.1017/S0144686X0000502X 11654434

[ref30] Kropf N. P. Tandy C. (1998). Narrative therapy with older clients: the use of a “meaning-making” approach. Clin. Gerontol. 18, 3–16. doi: 10.1300/J018v18n04_02

[ref31] Landau R. Werner S. (2012). Ethical aspects of using GPS for tracking people with dementia: recommendations for practice. Int. Psychogeriatr. 24, 358–366. doi: 10.1017/S1041610211001888, PMID: 22014284

[ref32] Lillekroken D. (2020). Slow nursing and its holistic place in dementia care: a secondary analysis of qualitative data from nurses working in nursing homes. Holist. Nurs. Pract. 34, 40–48. doi: 10.1097/HNP.0000000000000361, PMID: 31725099

[ref33] Lima J. C. Schwarz M. L. Clark M. A. Miller S. C. Degenholtz H. B. (2020). The changing adoption of culture change practices in US nursing homes. Innov. Aging 4:igaa012. doi: 10.1093/geroni/igaa012, PMID: 32529051 PMC7272786

[ref34] Lincoln Y. S. Guba E. G. (1985). Naturalistic Inquiry. New Delhi, India, and Newbury Park, London: Sage Publications.

[ref35] Martela F. Ryan R. M. Steger M. F. (2018). Meaningfulness as satisfaction of autonomy, competence, relatedness, and beneficence: comparing the four satisfactions and positive affect as predictors of meaning in life. J. Happiness Stud. 19, 1261–1282. doi: 10.1007/s10902-017-9869-7

[ref36] McCormack B. (2004). Person-centredness in Gerontological nursing: an overview of the literature. J. Clin. Nurs. 13, 31–38. doi: 10.1111/j.1365-2702.2004.00924.x, PMID: 15028037

[ref37] McCormack B. (2010). Person-centeredness in gerontological nursing: an overview of the patients with Alzheimer’s disease as perceived by nursing students and supervising nurses. J. Clin. Nurs. 19, 2639–2648. doi: 10.111/j.1365-2702.2010.03190.x 20920084

[ref38] Ministry of Health and Care Services (2006). White Paper 25 (2005–2006). Mestring, muligheter og mening [Coping, Possibilities and Meaning]. Oslo.

[ref39] Ministry of Health and Care Services (2009). White Paper 47 (2008–2009). Samhandlingsreformen. Rett behandling – på rett sted – til rett tid [The Coordination Reform. Proper Treatment – At the Right Place – And the Right Time]. Oslo.

[ref40] Ministry of Health and Care Services (2018). White Paper 15 (2017–2018). Leve hele livet – en kvalitetsreform for eldre [Live Throughout Life – A Quality Reform for Older People]. Oslo.

[ref41] Mulgan G. (2007). Ready or Not? Taking Innovation in the Public Sector Seriously. NESTA Provocation 03.

[ref42] Narens L. (2002). Theories of Meaningfulness. Scientific Psychology Series. Irvine: University of California.

[ref43] Nordin S. McKee K. Wallinder M. von Koch L. Wijk H. Elf M. (2017). The physical environment, activity, and interaction in residential care facilities for older people: a comparative case study. Scand. J. Caring Sci. 31, 727–738. doi: 10.1111/scs.12391, PMID: 27862156

[ref44] Park C. L. (2010). Making sense of the meaning literature: an integrative review of meaning making and its effects on adjustment to stressful life events. Psychol. Bull. 136, 257–301. doi: 10.1037/a0018301, PMID: 20192563

[ref45] Pue K. Vandergeest C. Breznitz D. (2016). Toward a Theory of Social Innovation. Innovation Policy White Paper Series 2016–01. Munk School of Global Affairs. University of Toronto, Canada.

[ref46] Ramkissoon H. (2020a). COVID-19 place confinement, pro-social, pro-environmental behaviors, and residents’ wellbeing: a new conceptual framework. Front. Psychol. 11:2248. doi: 10.3389/fpsyg.2020.02248, PMID: 32982895 PMC7490327

[ref47] Ramkissoon H. (2020b). Perceived social impacts of tourism and quality-of-life: a new conceptual model. J. Sustain. Tour. 1–18. doi: 10.1080/09669582.2020.1858091

[ref48] Ramkissoon H. (2021). Place affect interventions during and after the COVID-19 pandemic. Front. Psychol. 12:726685. doi: 10.3389/fpsyg.2021.726685, PMID: 34594279 PMC8476834

[ref49] Ramkissoon H. Smith L. D. G. Weiler B. (2013). Testing the dimensionality of place attachment and its relationships with place satisfaction and pro-environmental behaviours: a structural equation modelling approach. Tour. Manag. 36, 552–566. doi: 10.1016/j.tourman.2012.09.003

[ref50] Ratwani R. M. (2017). Electronic health records and improved patient care: opportunities for applied psychology. Curr. Dir. Psychol. Sci. 26, 359–365. doi: 10.1177/0963721417700691, PMID: 28808359 PMC5553914

[ref51] Robinson G. Gallagher A. (2008). Culture change impacts quality of life for nursing home residents. Top. Clin. Nutr. 23, 120–130. doi: 10.1097/01.TIN.0000318908.08617.49

[ref52] Shotton L. Seedhouse D. (1998). Practical dignity in caring. Nurs. Ethics 5, 246–255. doi: 10.1177/096973309800500308 9653222

[ref53] Slettebø Å. Sæteren B. Caspari S. Lohne V. Rehnsfeldt A. Heggestad A. K. T.. (2017). The significance of meaningful and enjoyable activities for nursing home resident’s experiences of dignity. Scand. J. Caring Sci. 31, 718–726. doi: 10.1111/scs.12386, PMID: 27910119

[ref54] Statistics Norway (2020). Care Services. Available at: https://www.ssb.no/en/helse/statistikker/pleie (Accessed January 11, 2021).

[ref55] Travelbee J. (1971). Interpersonal Aspects of Nursing. 2nd Edn. Philadelphia, PA: F.A. Davis Company.

[ref56] Tronto J. C. (2001). “An ethic of care,” in Ethics in Community-Based Elder Care. eds. Holstein M. Mitzen P. (New York: Springer Publishing Company), 60–68.

[ref57] Wegener C. Tanggaard L. (2013). The concept of innovation as perceived by public sector frontline staff: outline of a tripartite empirical model of innovation. Stud. Contin. Educ. 35, 82–101. doi: 10.1080/0158037X.2012.707123

[ref58] Wong P. T. P. (2017). A decade of meaning: past, present, and future. J. Constr. Psychol. 30, 82–89. doi: 10.1080/10720537.2015.1119085

[ref59] World Medical Association (2013). World medical association declaration of Helsinki. Ethical principles for medical research involving human subjects. JAMA 310, 2191–2194. doi: 10.1001/jama.2013.281053, PMID: 24141714

